# Simulating Treating in Place in Long-Term Care: Interprofessional Team Care Video Case for Nurse Practitioners

**DOI:** 10.1093/geroni/igab046.2801

**Published:** 2021-12-17

**Authors:** Laurie Kennedy-Malone

**Affiliations:** UNCG School of Nursing, Greensboro, North Carolina, United States

## Abstract

As a means of enhancing clinical simulation opportunities for adult-gerontology nurse practitioner students, a series of video simulations were created for use for nurse practitioner education. With funding through the Health Resources and Service Administration (HRSA) Advanced Nursing Education Workforce grant and partnering with nurse practitioner clinical educators from Optum Health Care, a video simulation focused on the concept of treating an older veteran within a long-term care facility rather than transferring to the acute care setting was developed. The case Treating in Place: Nurse Practitioner-Led Team Management of a Long-Term Care Patient Video involved a nurse practitioner collaborating with a physician, a registered nurse, a social worker, and a family member. The interactive simulation video was developed using the eLearning authoring tool H5P to create learning experiences for students that can be used either in face-to-face classroom experiences or embedded in learning management systems. H5P is a web-based authoring tool that helps faculty build interactive course content. H5P activities provide instant feedback to students, allowing them to self-assess their understanding of the dynamic video simulation case. A faculty handbook that describes the case scenario with the interactive questions and suggested discussion questions is available. The adult-gerontology primary care nurse practitioner competencies addressed for this case are identified in the faculty handbook. These videos have been widely disseminated and are being included in nurse practitioner curriculum across the country. A QR code with access to direct viewing of the video will be included in the presentation.

